# Methoxylation enhances stilbene bioactivity in *Caenorhabditis elegans*

**DOI:** 10.1186/1471-2210-8-15

**Published:** 2008-08-13

**Authors:** Mark A Wilson, Agnes M Rimando, Catherine A Wolkow

**Affiliations:** 1 Laboratory of Neurosciences, National Institute on Aging, Intramural Research Program, NIH, Baltimore, MD 21224, USA; 2 Natural Products Utilization Research Unit, ARS, US Department of Agriculture, PO Box 8048, University, MS 38677, USA

## Abstract

**Background:**

Stilbenes are 1,2-diphenylethylene congeners produced by plants in response to stress. Many stilbenes also exhibit xenobiotic activities in animal cells, such as inhibition of cancer cell growth, neuroprotection, and immune modulation. *In vivo*, hydroxylated stilbenes are metabolized by glucuronidation to facilitate excretion. Methoxylated stilbenes are metabolized more slowly, which may have a positive effect on *in vivo *bioactivity. Here, we have directly compared *in vivo *bioactivities of methoxylated and hydroxylated stilbenes in a whole organism using the roundworm *Caenorhabditis elegans*, an advantageous experimental system for such studies due to its rapid lifecycle, genetic amenability and relatively low-cost.

**Results:**

Toxicity towards *C. elegans *adults was observed for trimethoxylated and dimethoxylated stilbenes, as well as the monomethoxylated stilbene desoxyrhapontigenin. Toxicity was not observed for the monomethoxylated stilbene, pinostilbene, nor for hydroxylated stilbenes. The methoxylated stilbenes that exhibited toxicity also showed stronger inhibitory effects than the hydroxylated stilbenes on germline tumor growth in *gld-1(q485) *adults. However, steady-state levels of three inhibitory methoxylated stilbenes did not directly correlate to their relative bioactivities.

**Conclusion:**

These findings demonstrate that, for the group of stilbenes investigated, methoxylation generally increased bioactivity *in vivo *in a whole organism, with the exception of pinostilbene. Differences in bioactivity in *C. elegans *adults did not appear to correlate with differential uptake. Rather, we speculate that methoxylated stilbenes may have increased interactions with biological targets *in vivo *or may interact with specific targets unaffected by hydroxylated stilbenes. The potent activities of methoxylated stilbenes provide a basis for further investigations to identify *in vivo *targets for these compounds.

## Background

Phytochemicals induce an array of biological responses in animal cells, including enzyme inhibition, protection against oxidation and regulation of cellular signaling pathways. Among phytochemicals with xenobiotic activities, the stilbenes are of great interest for their potential medical impact. Stilbene compounds, particularly pterostilbene and resveratrol, have been associated with antidiabetic, anticarcinogenic and antilipogenic activities *in vitro *and *in vivo *[1-4]. Specific enzymatic targets are known for some of these xenobiotic activities. Pterostilbene and related compounds inhibit the cytochrome P450 enzymes, CYP1A1 and CYP1B1, which induce carcinogenicity of environmental teratogens [5]. Pterostilbene also inhibits cyclooxygenases COX-1 and COX-2, interfering with endocrine functions and supporting analgesic activity [6]. Significant increase in activity of hexokinase, while significant decrease in activity of glucose-6-phosphatase and fructose-1,6-bisphosphatase, were observed after oral administration of pterostilbene in diabetic rats [7]. The related compound, resveratrol, stimulates sirtuin enzymes to promote longevity in response to dietary restriction [8,9]. Resveratrol has also been demonstrated to protect against obesity- and diet-related disease in rodents [10]. Additional interactions with new targets may be found to underlie other activities of these compounds.

While dietary stilbenes and flavonoids are of particular interest for their potential health benefits, *in vivo *activity is limited by low bioavailability due to rapid metabolism and excretion. Studies have found that methoxylation can protect flavonoids from derivatization, thereby improving biostability [11,12]. However, there is also evidence that methoxylation can alter bioactivity. For example, methoxylated flavones induced different cell-cycle arrest points in cultured human cells than hydroxylated flavones [13]. Thus, methoxylation may alter interactions with target proteins, as well as increase bioavailability. These reports on altered bioactivity in methoxylated flavonoids triggered our interest to investigate the effects of methoxylation on stilbene bioactivity, particularly in a whole organism, to assess effects on postmitotic cells in adult tissues as well as on actively dividing cells.

Use of invertebrates provides one approach to address these questions rapidly and economically in a whole organism. In particular, the roundworm, *Caenorhabditis elegans*, offers many advantages for such analyses. *C. elegans *has a short generation time of 1 week and are easily maintained in large numbers with ordinary laboratory equipment. The adult body is approximately 1 mm in length, and contains most of the tissues present in higher vertebrates, such as a complete nervous system, striated muscles and an intestine with digestive, detoxification and innate immune functions. It is of note that the adult somatic tissues are post-mitotic and therefore lack cell-replacement capacity. *C. elegans *are hermaphrodites and able to self-fertilize, which has greatly facilitated the genetic analysis of developmental and cell biological processes in this organism. Previous uses of *C. elegans *for pharmacological studies have taken advantage of mutant strains for mechanistic studies, in addition to basic studies of compound-induced gene expression or phenotypic changes [14-16].

In our study, we used *C. elegans *to compare the bioactivities of methoxylated and hydroxylated stilbenes, based on the parent compound resveratrol. Seven stilbenes differing in hydroxylation and methoxylation patterns were examined for effects on survival of *fem-1(hc17) *sterile adults and growth of germline tumors in *gld-1(q485) *mutants. Methoxylated stilbenes exhibited greater bioactivity in both assays, as compared with hydroxylated stilbenes. Steady-state levels for three of these compounds (one mono-, one di-, and one tri-methoxylated) were measured in treated worms and were not found to correlate with relative bioactivity. This suggests that the greater bioactivity of methoxylated stilbenes does not reflect differential uptake of the compounds. These findings demonstrate that methoxylation substitution enhances stilbene bioactivity in this whole-organism model. Methyl group-specific target site interactions may be one factor accounting for the differential bioactivities of these compounds. Alternatively, methoxylation may protect stilbenes from metabolic modification and excretion, leading to higher potency *in vivo *in *C. elegans *than hydroxylated stilbenes.

## Results and discussion

**Figure 1:**
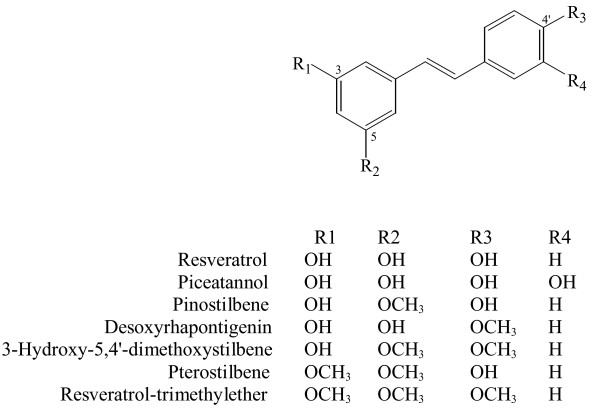
Structures of methoxylated and hydroxylated stilbenes tested in this study.

Seven methoxylated or hydroxylated stilbenes were compared for effects on *C. elegans *survival and tumor growth. This collection included two hydroxylated structures (resveratrol and piceatannol), two monomethoxylated structures (pinostilbene and desoxyrhapontigenin), two dimethoxylated structures (3-hydroxy-5,4'-dimethoxystilbene and pterostilbene) and one trimethoxylated structure (resveratrol-trimethylether) (Figure 1). Bioactivity in *C. elegans *has been reported only for resveratrol, which extended adult lifespan [9]. It was of interest, therefore, to compare adult survival in animals treated with resveratrol and the related compounds.

For survival assays, young adult (day 0) animals were transferred onto fresh medium supplemented with the test compound at a range of concentrations. The compounds were tested at indicated concentrations in the growth medium (below). *C. elegans *nematodes are surrounded by a thick collagenous cuticle and are therefore relatively impermeable to environmental compounds. Consistent with this, our measurements demonstrated that internal concentrations of these compounds were significantly lower than their concentrations in the medium. Survival was scored every 2–3 days as the ability to move in response to a gentle mechanical stimulus. During the first week of adulthood, *C. elegans *hermaphrodites are reproductively competent and normally produce 200–300 progeny. Since progeny production would complicate survival analysis, these experiments were performed using *fem-1(hc17) *animals that are sterile due to a spermatogenesis defect [17,18]. Our prior analyses have confirmed that *fem-1(hc17) *and wildtype animals have similar lifespan characteristics [15].

**Figure 2:**
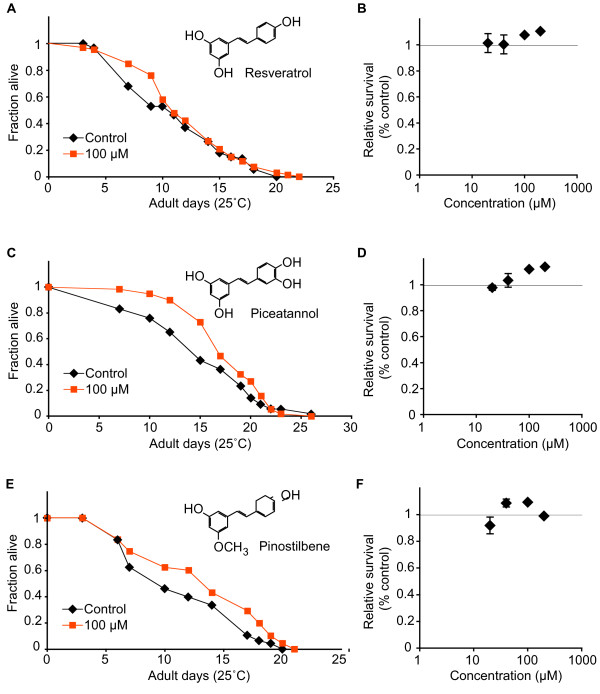
**Hydroxylated and one monomethoxylated stilbene had modest beneficial effects on adult *C. elegans *survival**. (A, C, E) Representative survival curves of adult sterile *fem-1(hc17) *animals treated with indicated doses of each compound or vehicle in the medium. Experiments for each compound were conducted concurrently for all doses shown. (B, D, F) Summary data from n = 1–3 independent experiments per concentration tested, showing dose-response for each compound. Complete survival statistics are presented in Table 1.

Under our experimental conditions, both hydroxylated and one monomethoxylated stilbene, pinostilbene, had negligible or modestly beneficial effects on adult survival (Figure 2). Consistent with previous reports, we observed that resveratrol had a modestly beneficial effect on adult lifespan (0–15% at 100 μM dose) (Table 1). However, the effect was variable and did not attain statistical significance in our experiments. In addition, piceatannol and pinostilbene had beneficial effects on lifetime survival. The effects of these compounds were also modest (5–15%) and were not statistically significant.

**Table 1:** Adult survival statistics for all trials.

**Trial**	**Concentration (μM)**	**Mean lifespan (days)**		**Relative survival (%)**	***p *(Logrank)**	**n (failed, censor)**
					
		**Treated**	**Control**			
***Resveratrol-trimethylether***						

1	100	9.7	12.16	80%	0.004	61,0
4	20	7.72	11.26	69%	< 0.001	61,0
4	40	7.72	11.26	69%	< 0.001	74,0
4	100	7.18	11.26	64%	< 0.001	70,2
5	5	18.83	18.49	102%	0.91	46,3
5	20	16.41	18.49	89%	0.055	42,3
5	100	15.18	18.49	82%	0.005	37,6
6	20	9.02	15.32	59%	< 0.0001	49,5
6	40	7.98	15.32	52%	< 0.0001	51,0
6	100	7.54	15.32	49%	< 0.0001	52,0

***3-OH-5,4'- dimethoxystilbene***						

1	100	6.34	12.16	52%	< 0.0001	53,0
2	20	11.05	11.05	100%	0.986	47,2
2	40	10.52	11.05	95%	0.234	40,1
6	20	10.38	15.32	68%	< 0.0001	56,0
6	40	7.68	15.32	50%	< 0.0001	54,1
6	100	6.56	15.32	43%	< 0.0001	55,3

***Pterostilbene***						

1	100	9.55	12.16	79%	0.005	69,9
4	20	10.43	11.26	93%	0.336	54,0
4	40	10	11.26	89%	0.035	65,1
4	100	8.07	11.26	72%	< 0.0001	51,1
6	20	13.72	15.32	90%	0.058	55,1
6	40	14.41	15.32	94%	0.142	51,4
6	100	10.98	15.32	72%	< 0.001	51,0

***Desoxyrhapontigenin***						

1	100	10.64	12.16	88%	0.12	61,1
3	100	11.31	15.37	74%	< 0.0001	36,0
3	200	11.61	15.37	76%	< 0.0001	44,1
4	20	10.2	11.26	91%	0.12	74,0
4	40	10.38	11.26	92%	0.11	71,2
4	100	9.44	11.26	84%	0.003	50,0
6	20	14.18	15.32	93%	0.494	54,3
6	40	15.31	15.32	100%	0.48	59,1
6	100	13.63	15.32	89%	0.027	46,9

***Pinostilbene***						

1	100	13.6	12.16	112%	0.062	68,1
2	20	10.04	11.05	91%	0.266	55,0
2	40	11.53	11.05	104%	0.319	42,5
3	100	16.9	15.37	110%	0.448	41,0
3	200	15.23	15.37	99%	0.411	42,5
6	20	14.24	15.32	93%	0.319	56,3
6	40	17.33	15.32	113%	0.085	60,0
6	100	16.24	15.32	106%	0.724	51,2

***Resveratrol***						

1	100	12.18	12.16	100%	0.867	70,3
2	20	11.85	11.05	107%	0.182	48,4
2	40	10.53	11.05	95%	0.924	55,0
3	100	16.64	15.37	108%	0.435	30,4
3	200	17	15.37	111%	0.337	38,0
6	20	14.65	15.32	96%	0.804	54,5
6	40	16.17	15.32	106%	0.501	59,0
6	100	17.55	15.32	115%	0.055	58,0

***Piceatannol***						

2	20	11.72	11.05	106%	0.362	44,0
2	40	11.58	11.05	105%	0.5	37,9
3	100	17.78	15.37	116%	0.094	55,1
3	200	17.5	15.37	114%	0.166	55,1
6	20	13.71	15.32	89%	0.231	50,1
6	40	15.63	15.32	102%	0.75	50,1
6	100	16.6	15.32	108%	0.146	50,1

**Figure 3:**
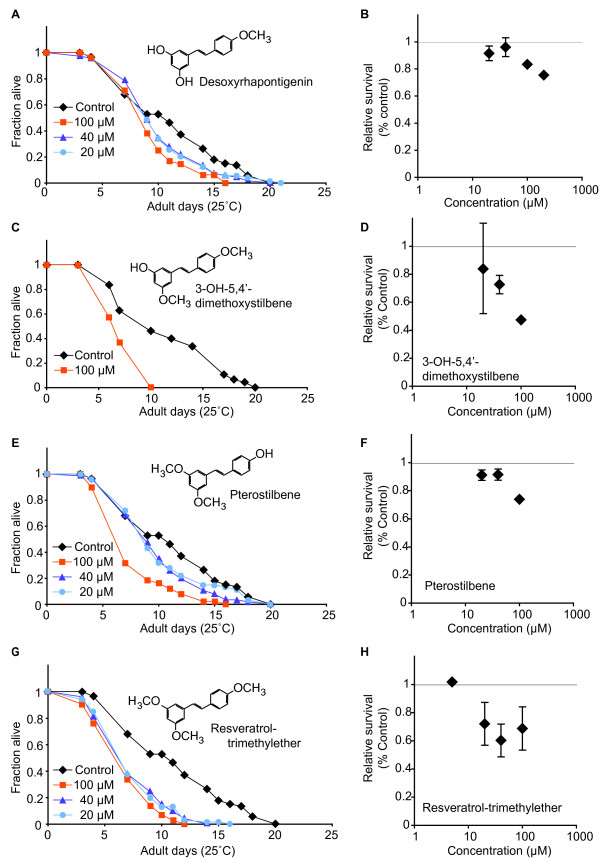
**Methoxylated stilbenes were toxic to *C. elegans *adults**. (A, C, E, G) Representative survival curves of *fem-1(hc17) *sterile adults treated with compounds as indicated from the first day of adulthood. (B, D, F, H) Summary data for independent experiments at indicated concentrations. Complete survival statistics presented in Table 1.

The methoxylated stilbenes, desoxyrhapontigenin, 3-hydroxy-5,4'-dimethoxystilbene, pterostilbene and resveratrol-trimethylether, each exhibited significant detrimental effects on adult survival (Figure 3). At a 100 μM dose initiated on the first day of adulthood, survival was reduced 11–26% by desoxyrhapontigenin, 48–57% by 3-hydroxy-5,4'-dimethoxystilbene, 21–28% by pterostilbene and 18–51% by resveratrol-trimethylether (Table 1). The toxicity of resveratrol-trimethyether increased steeply within a relatively limited dose range, between 5–20 μM. In contrast, the toxicity of desoxyrhapontigenin, 3-hydroxy-5,4'-dimethoxystilbene and pterostilbene gradually increased between the 20–100 μM doses. The observed difference in activity between the monomethoxylated stilbenes, desoxyrhapontigenin being toxic while pinostilbene not, may be due to differences in steric hinderance introduced by the bulky methoxy groups at different positions in the stilbene ring. Without knowing the target site(s) in *C. elegans *for these stilbenes, it can be speculated that the position of the methoxy group at ring A in pinostilbene causes unfavourable fit for target site inhibition. Differences in activity of these monomethoxylated stilbenes have been demonstrated in other studies; *e.g*., pinostilbene moderately inhibited the catalytic activity of cytochrome P450 2E1, while desoxyrhapontigenin was not inhibitory [19]. We noted that animals treated with either methoxylated or hydroxylated stilbenes did not display any other noticeable phenotypes, such as altered feeding or movement rates.

**Figure 4:**
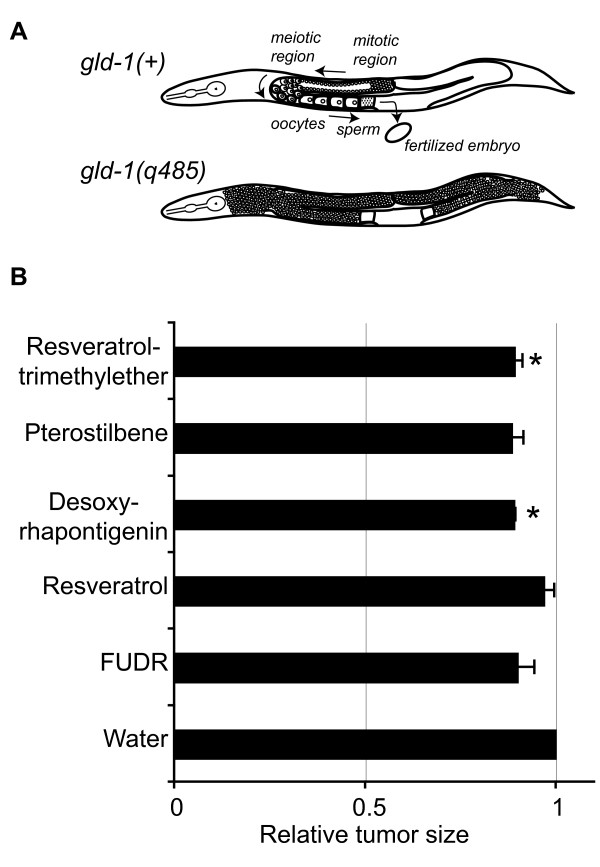
**Methoxylated stilbenes, desoxyrhapontigenin and resveratrol-trimethyether, demonstrated modest anti-tumor activity *in vivo***. (A) Upper right, cartoon of normal germ cell proliferation and differentiation in *gld-1(+) *animals. Below, cartoon of aberrant germline tumors formed in *gld-1(q485) *animals. In tumor-containing *glp-1(q485) *adults, DAPI staining allows visualization and quantification of germline tumors as the fluorescent area filling the body. (B) Mean germline tumor size (area ± SEM) relative to control in *glp-1(q485) *adults treated with 100 μM doses of indicated stilbenes or FUDR. Results are average from at least 3 independent experiments. In each experiment, tumor area was determined for 12–47 individuals per compound (average ± SD = 23.1 ± 10.12 animals). Significance was judged in paired t-test (2-tailed); * *p *< 0.05 versus untreated.

The somatic tissues of *C. elegans *adults are comprised solely of post-mitotic cells and lack the ability to renew by stem cell replacement. To more closely examine the effects of methoxylated stilbenes on mitotically-active cells in this organism, we examined their effects on growth of germline tumors produced in *gld-1(q485) *animals. The *C. elegans *hermaphrodite gonad is a U-shaped structure that is bisymmetric and contains the germ cell pool (Figure 4A, *gld-1(+)*). Within each gonad arm, germ cells in the distal region undergo mitotic proliferation in response to *Notch*-like signals produced by cells at the distal tips of the gonad arms. As germ cells migrate along the gonad arm, they exit from mitosis, undergo meiosis and subsequently differentiate into mature oocytes. In *gld-1(q485) *mutants, the germ cells fail to exit from mitosis and continue to proliferate throughout the gonad forming a germline tumor that is lethal to the animal (Figure 4A, *gld-1(q485)*) [20,21].

Several stilbenes have demonstrated antiproliferative effects on mammalian cancer cells *in vitro *[22-25]. To assess the effects of methoxylation on antitumor activity *in vivo*, tumor area was measured in *gld-1(q485) *adults treated for four days with either resveratrol trimethylether, desoxyrhapontigenin or pterostilbene, which are methoxylated and toxic to adults, or resveratrol, which is hydroxylated and nontoxic. As controls, germline tumor area was also measured in animals treated with carrier only (ethanol) or with 5-fluoro-2'-deoxyuradine (FUDR), a thymidylate synthetase inhibitor that blocks cell proliferation [26,27]. FUDR was chosen as a positive control for its known antiproliferative effects in *C. elegans*, which have been demonstrated by inhibition of embryonic and larval development [28]. After four days of treatment, animals were collected and germline tumors were visualized by DAPI staining to visualize germcell nuclei. Because the level of DAPI staining was not quantitative between animals, we DAPI signal intensity was not used as a measure of tumor size. Rather, tumor size was directly measured as the area of the DAPI-stained gonad.

For the initial tests of tumor growth suppression, each compound was tested at a 100 μM dose in the growth medium (Figure 4B). At this concentration, FUDR exhibited a modest inhibitory effect on *glp-1(q485) *germline tumor growth in two of three experiments (-4%, -7%, -19%; *p *≤ 0.05 for trials 2, 3, t-test vs untreated). At 100 μM dose, the methoxylated stilbenes resveratrol trimethylether and desoxyrhapontigenin were also associated with consistent reductions in tumor size. Pterostilbene also modestly reduced tumor size, and this effect was statistically significant in 2 of 3 trials (*p *≤ 0.05, t-test vs untreated). In contrast, resveratrol had a negligible effect on *gld-1(q485) *germline tumor size which was not statistically significant (-4%, *p *= 0.35). As expected from its known antiproliferative effect, FUDR treatment also appeared to decrease tumor size, although the effect was statistically significant in only 2 of 3 trials.

**Figure 5:**
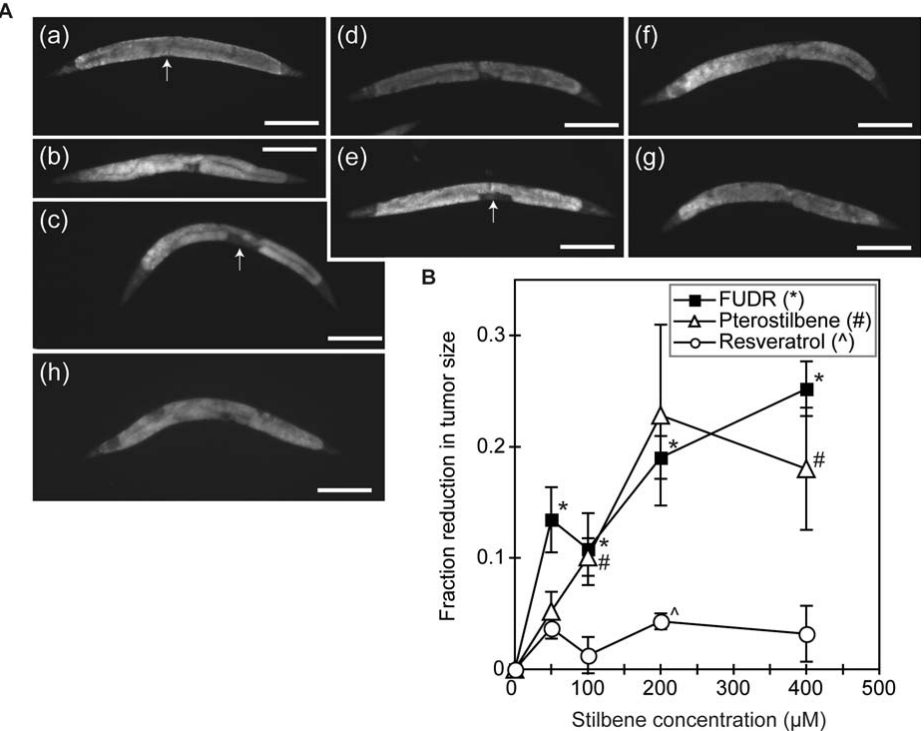
**Titration of germline tumor growth suppression by FUDR, resveratrol and pterostilbene**. (A) Representative images of DAPI-stained *glp-1(q485) *animals treated for four days with 100 μM or 400 μM doses of FUDR, resveratrol and pterostilbene. The inhibitory effect of FUDR and pterostilbene on tumor size is particularly evident near the central region of the animal where the reflexed arms of the distal gonad meet (arrow in (a), (c) and (e)). (a, h) vehicle-treated controls. (h) is from the same experimental series as (c); (a) is from the same experimental series as other images; (b, c), FUDR at 100 μM (b) and 400 μM (c); (d, e), pterostilbene at 100 μM (d) and 400 μM (e); (f, g) resveratrol at 100 μM (f) and 400 μM (g). Scale bar = 250 μM. (B) Summary of results from three independent experiments for each compound at indicated doses. Each concentration was tested in at least 3 independent trials and a total of 36–100 animals were measured per compound dose in all experiments. Significance was judged by a paired t-test (2-tailed); *, #, ^ *p *< 0.05 versus untreated for FUDR-, pterostilbene- or resveratrol-treated animals, respectively.

To better quantify the effects of stilbenes and FUDR on *gld-1(q485) *tumor growth, two stilbenes, resveratrol and pterostilbene, and FUDR were examined at a series of additional doses between 50–400 μM. Over this range of concentrations, both FUDR and pterostilbene were associated with 10–20% reduction in tumor area (Figure 5A, B). The effects of pterostilbene were somewhat variable between trials, and were statistically significant for 3 of 4 experiments at both the 200 and 400 μM dose. The 200 and 400 μM doses of FUDR were consistently associated with smaller tumor area and these effects were significant in all 4 trials. Resveratrol at the 200 and 400 μM dose was not associated with statistically significant reductions in tumor area for any of the three trials conducted. These data demonstrate that the enhanced bioactivity of methoxylated stilbenes *in vivo *in *C. elegans *can be detected as a reduction in *glp-1(q485) *tumor area as well as by adult toxicity.

**Figure 6:**
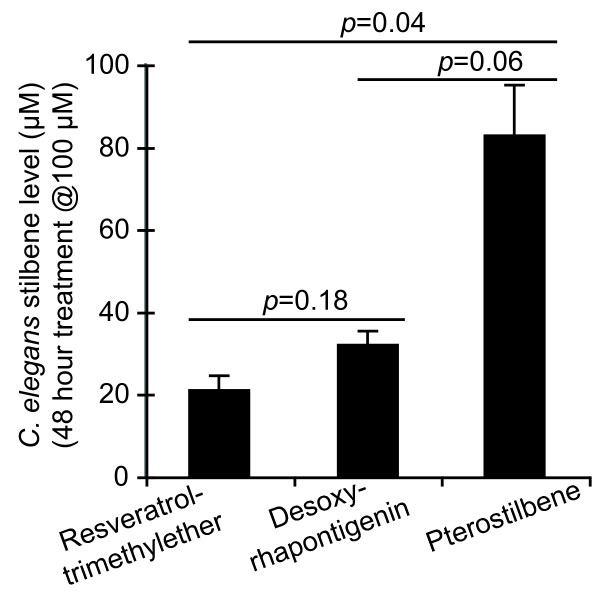
**Steady-state levels of three stilbene compounds in treated *C. elegans *adults did not correlate with toxicity**. Animals were treated for two days with a toxic dose (100 μM) of indicated compound in the medium. After collection, stilbene levels determined as described in methods. Compounds were measured in two independent samples for each treatment with paired negative control. Averages and SDs between both measurements are shown; statistical significance was determined by t-test.

The differential bioactivities of methoxylated and hydroxylated stilbenes in *C. elegans *adults might reflect differences in uptake or bioavailability. In general, intact *C. elegans *adults take up compounds relatively poorly due to their thick, impermeable cuticle. This impermeability has impeded pharmacodynamic analysis in this organism. However, steady-state levels of compounds can be measured in treated animals to provide one measure of pharmacodynamics. To assess whether differential uptake correlated with bioactivity in *C. elegans*, steady-state levels for pterostilbene, resveratrol-trimethylether and desoxyrhapontigenin were determined in animals treated for 2 days with 100 μM of each compound in the culture medium. The levels of these stilbenes in *C. elegans *nematodes ranged from 20–80 μM, indicating relatively efficient uptake (Figure 6). These concentrations were within the range of serum stilbene levels detected after dietary stilbene supplementation with human volunteers (approx. 2 μM) and rabbits (42.8 μM) [29]. It is also within the dose range where resveratrol was observed to induce dilation of isolated retinal arterioles (50 μM) [30]. These three compounds, while all methoxylated, exhibited different levels of toxicity in adult *C. elegans*. In treated worms, levels of pterostilbene were slightly elevated compared with resveratrol-trimethylether and desoxyrhapontigenin (Figure 6). However, the most toxic compound in this group, resveratrol-trimethylether, was present at less than one-third the level of pterostilbene. These data suggest that relative bioactivity did not solely reflect steady-state levels in intact animals, although, this experiment does not directly compare the pharmacokinetic profile of each compound in *C. elegans*.

This study compared the effects of methoxylated and hydroxylated stilbenes on adult lifespan and tumor growth in *C. elegans *nematodes. In this organism, stilbene methoxylation was associated with toxicity under chronic treatment conditions and reduced tumor size *in vivo*. Overall, this study corroborated other findings of enhanced bioactivities in methoxylated versus hydroxylated stilbenes in other experimental systems [31]. However, ours is the first study to systematically evaluate the effects of stilbene methoxylation on bioactivity in a whole organism, using *C. elegans*.

The mechanisms for enhanced bioactivity of methoxylated stilbenes may reflect increased biostability *in vivo *due to reduced efficiency of metabolic modification leading to excretion. In the case of another group of compounds, the flavonoids, substituting the hydroxyl groups with methoxy groups was shown to improve flavonoid stability in cultured cells and liver microsomes by blocking metabolic modification and excretion [12,11]. In mammals, hydroxylated flavonoids were shown to undergo glucuronidation by hepatic enzymes to promote excretion [11]. Thus, methoxylation may block this modification and may also promote biostability. Indeed, one naturally methoxylated stilbene, pterostilbene, is cytotoxic in several cellular systems [32]. In contrast, the hydroxylated stilbene, resveratrol, has also been shown to be cytotoxic, but is poorly retained in plasma compared with pterostilbene [33]. Thus, methoxylation differences between pterostilbene and resveratrol may improve bioactivity by increasing *in vivo *stability.

The basis for the toxicity and antiproliferative activities of methyoxylated stilbenes in *C. elegans *adults is not known. The somatic cells of *C. elegans *adults are entirely postmitotic, ruling out antiproliferative effects as the basis for methoxylated stilbene toxicity. However, the toxicity and antitumor activities of methoxylated stilbenes in *C. elegans *adults may reflect interactions with common biological targets. Studies in other experimental systems show that stilbenes are potent antioxidants [34,35]. However, resveratrol and pterostilbene, which have strikingly different activity *in vivo *in adult *C. elegans*, demonstrated similar antioxidant activity *in vitro*, suggesting that antioxidant activity is not the underlying reason for the difference in stilbene bioactivities in *C. elegans*. Stilbenes are also known to inhibit specific cellular enzymes, such as cytochrome P450 and COX enzymes. One study has compared the relative inhibition of mammalian CYP1 cytochrome P450 enzymes by pterostilbene, pinostilbene and desoxyrhapontigenin [5]. In this study, these three methoxylated stilbenes demonstrated increased CYP1 inhibition relative to resveratrol, in partial agreement with our results. However, pinostilbene demonstrated slightly stronger CYP1 inhibition than pterostilbene and desoxyrhapontigenin, a finding not consistent with our *C. elegans *toxicity data. Stilbenes also inhibit COX-1 and -2 monooxygenases. *C. elegans *nematodes do not have any obvious COX-1 or -2 homologs, although the *C. elegans *genome sequence does contain numerous monooxygenases that might have similar mechanisms of action to mammalian COX-1 and -2 enzymes. Further characterization of the *in vivo *targets for methyoxylated stilbenes in *C. elegans *may provide insight into other bioactivities of these compounds in human cells.

## Conclusion

Stilbene methoxylation was associated with increased *in vivo *bioactivity as tested by toxicity and tumor growth in *C. elegans*. In general, increasing degree of methoxylation was correlated with increasing bioactivity, with the exception of pinostilbene. However, methoxylation and toxicity were not correlated with steady-state levels of these compounds in treated animals. These findings suggest that methoxylation enhances bioactivity possibly through increased interactions with *in vivo *targets. A working hypothesis to account for these results is that stilbene methoxylation protects the compound from conjugation and subsequent excretion, thereby increasing biostability and bioavailability in *C. elegans*.

## Methods

### *C. elegans *strains and culture conditions

*C. elegans *strains were maintained at 15°C on NGM agar medium with a live, slow-growing OP50 bacterial lawn for a food source following standard protocols [36]. The following mutant strains were used in this study: BA17, *fem-1(hc17)*, and JK1466, *gld-1(q485)/dpy-5(e61) unc-13(e51)*. Strains were obtained from the *Canorhabditis *Genetics Center at the University of Minnesota.

### Chemical sources and methoxylated stilbene preparation

Resveratrol and picetannol were obtained from commercial sources (Sigma-Aldrich, Inc. and Calbiochem-Novobiochem Corp., respectively). The methoxylated stilbenes pinostilbene, desoxyrhapontigenin, 3-hydroxy-5,4'-dimethoxystilbene and pterostilbene (Figure 1) were synthesized by partial methylation of *trans*-resveratrol as previously reported [34]. The methoxylated stilbenes were purified by preparative layer chromatography (Merck Silica gel 60 F254; EMD Chemicals Inc., Gibbstown, NJ) using the developing solvent chloroform:methanol (96:4) with 1% formic acid. The Rf values for pterostilbene, 3-hydroxy-5,4'-dimethoxystilbene, pinostilbene and desoxyrhapontigenin are 0.61, 0.57, 0.28, and 0.20, respectively. These stilbenes were identified from their ^1^H-NMR spectra (Bruker 400 MHz; Bruker, Billerica, MA). All stilbenes were dissolved in ethanol to 25 mg/mL before use. 5-Fluoro-deoxyuracil (Sigma Chemical Co.) was dissolved to 1 mg/mL in sterile water and stored frozen at -20°C until use. For treating *C. elegans *adults, each compound was added in a 200 μL volume to the top of a 1-day old bacterial lawn grown on NGM medium to final concentrations as indicated. Compounds were allowed 2 hours to diffuse through the agar medium before animals were added.

### Survival assays

For survival analyses, synchronized populations of sterile *fem-1(hc17) *hermaphrodites were obtained by first transferring several fertile hermaphrodites to a fresh plate at 25°C and allowing them to lay eggs for several hours. The parent hermaphrodites were removed and embryos were allowed to develop into sterile adults at 25°C, which takes approximately 48 hours [17]. On the first day of adulthood, referred to as day 0, sterile adults were transferred onto fresh media supplemented with stilbenes at indicated concentrations, and maintained at 25°C. Adult survival was scored every 2–3 days by the animal's ability to move in response to a gentle mechanical stimulus. Statistical analysis of survival was performed using the JMP software package.

### Germline tumor growth assays

The *gld-1(q485) *mutation causes a defect in oocyte development that results in growth of germline tumors that fill the somatic gonad, eventually leading to the animal's death [20,21]. To examine the effects of the stilbenes on *gld-1(q485) *germline tumors, day 0 adults were placed on stilbene-containing medium in the presence of OP50 bacteria as food source. After 4 days at 20°C, animals were fixed in methanol and germcell nuclei were visualized by staining with DAPI. Images were collected with the entire animal in view using the 4× objective on a Nikon E800 microscope equipped with a Hamamatsu Orca ER CCD camera using OpenLab imaging software. NIH Image J software was used to measure germline tumor growth in treated and untreated animals. Tumor area was measured by outlining the entire DAPI-stained tumor.

### Determination of stilbene uptake

Uptake was determined for three stilbenes (desoxyrhapontigenin, pterostilbene and resveratrol-trimethylether) by measuring the steady-state levels of each stilbene in adult *fem-1(hc17) *hermaphrodites treated under the same conditions as those used for survival assays. After a 2-day treatment on stilbene-supplemented medium, animals were collected into eppendorf tubes, washed with sterile M9 and allowed to settle under a minimal volume of liquid. The samples were frozen at -80°C for storage. For the analysis of stilbenes, samples were thawed in ice and homogenized in 300 μL of 0.2 M phosphate buffer then centrifuged (4°C, 4000 rpm, 15 min). The supernatant was collected and the pellet was homogenized further with fresh 300 μL of 0.2 M phosphate buffer then centrifuged. The supernatants were combined and loaded on an Oasis^® ^HLB solid phase extraction cartridge (Waters Corporation, Milford, MA) that has been preconditioned with 0.2 M phosphate buffer. The stilbenes were eluted with 2 mL of methanol and collected in a vial. The eluate was dried under a stream of nitrogen, redissolved in 1 mL of ethyl acetate, filtered (0.2 μm nylon filter) then dried again under a stream of nitrogen. The dried sample was treated with 30 μL of N,O-*bis *[trimethylsilyl]trifluoroacetamide:dimethylformamide (BSTFA: DMF, 1:1; Pierce Biotechnology, Inc., Rockford, IL) and heated at 70°C for 45 min. Derivatized sample was analyzed for stilbenes by gas chromatography-mass spectrometry (GC-MS). GC-MS was performed on a JEOL GCMate II spectrometer (JEOL USA Inc., Peabody, MA) in tandem with an Agilent 6890N gas chromatograph (Agilent Technologies, Santa Clara, CA) using a J&W DB-5 capillary column (0.25 mm internal diameter, 0.25 μm film thickness, 30 m length; Agilent Technologies). The GC temperature program was: initial 190°C, increased to 240°C at 25°C/min rate and held at this temp for 14 min, then finally increased to 300°C at the rate of 25°C/min and held at this temperature for 1 min. The carrier gas was ultrahigh purity helium, at 1 mL/min flow rate. The injection port, GC-MS interface and ionization chamber were at 250, 230 and 230°C, respectively. The volume of injection was 1 μL, splitless injection. Mass spectrum was acquired in positive, electron impact (70 eV), selected ion monitoring mode. Resveratrol-trimethylether (retention time 10.6 min) was monitored at *m/z *270, 239 and 196; desoxyrhapontigenin (retention time 13.1 min) was monitored at *m/z *328, 313 and 206; pterostilbene (retention time 13.6 min) was monitored at *m/z *386, 371 and 297. Quantitation was performed from calibration curve of authentic stilbene samples. GC-MS analyses were in duplicates. For conversion from pg/worm to molarity concentration *in vivo*, the volume of a wildtype hermaphrodite on day 4 of adulthood was taken to be 4.5 μL, as previously determined [37].

## Authors' contributions

MAW and CAW conceived of and designed the *C. elegans *experiments, which were performed entirely by MAW. AMR prepared the stilbene derivatives and performed the uptake determinations. CAW, MAW and AMR prepared the manuscript. All authors have read and approved the manuscript.
